# American Medical Association at Detroit

**Published:** 1892-07

**Authors:** 


BUFFALO MEDICAL AND SURGICAL JOURNAL

A MONTHLY REVIEW OF MEDICINE AND SURGERY.

*EDITORS:*

THOMAS LOTHROP, M. D. - - WM. WARREN POTTER, M. D.

All communications, whether of a literary or business character, should be addressed to the managing editor: 284 Franklin Street, Buffalo, N. Y.

Vol. XXXI. JULY, 1892. No. 12.

### AMERICAN MEDICAL ASSOCIATION AT DETROIT.

The forty-third annual meeting of this Association, held in Detroit, June 7, 8, 9, and 10, 1892, was one of the most remarkable in its history. The location was a favorable one for a large attendance, and the expectations of the officers in this regard were fully realized. The month of June is an attractive one for such a meeting, and the registration numbered something over 1,100. The invited guests, exhibitors, ladies, and attachés swelled this to an aggregate of fully 1,500 visitors, all of whom the hospitable city of the convention provided for in a most acceptable manner. The Committee of Arrangements and the various sub-committees were kept busy for weeks in preparation for the meeting, and their endurance was taxed in a high degree during the sessions. The social part of the programme was an unusually good one, and the citizens of Detroit who contributed to the pleasure and comfort of the visitors have nothing to regret.

The dinner to the Medical Editors, given by Mr. George S. Davis, at the Detroit Club on Monday, June 6th, was one of the most elegant that has been prepared for that Association.

The exhibit hall was well filled with elegant pharmaceutical, mechanical, and literary productions, and was one of the most satisfactory collections of these several departments that we have ever seen.

Messrs. Parke, Davis & Co. fairly outdid themselves in the royal manner in which they entertained all who visited their great laboratory, which is the most extensive plant of its kind in the world. Especially do we recall the pleasant greeting that was given by the chiefs of their several bureaus, who received and conducted the visitors in groups through their respective departments. Their private fire department was turned out time and time again, and their military band discoursed agreeable music in the court to hundreds of amazed and gratified people. Whoever inspected this wonderful establishment could not fail to depart with a confidence that this famous house was manufacturing and sending out an elegant line of pharmaceutical preparations, that were just precisely what on their face they claimed to be.

The sections performed an enormous amount of scientific work, as attested by the reading and the discussions of over 400 papers, most of which were of a high order, and will be a substantial contribution to the literature of medicine for the year 1892.

The general meetings were very largely attended, and an unusual interest was manifested in the controversy that arose with reference to the code question, which was revived with all the old fire on the part of some of the leaders of the Association, who are the defenders of the so-called old code. The Association was only prevented from committing itself to its former intolerant attitude on this question by a sturdy phalanx of able men, led by Dr. Dudley S. Reynolds, of Kentucky; Dr. A. L. Gihon, of the U. S. Navy; Dr. Charles A. L. Reed, of Cincinnati; Dr. Lewis S. McMurtry, of Kentucky, and Dr. W. P. King, of Missouri. These were supported by many able coadjutors, whom we have not space to mention. If it is admitted that the old code group triumphed in one respect, namely, in displacing one poor little trustee, it must also be admitted that progress was made in another way that two or three years ago would have been thought impossible. When the American Medical Association proposed the appointment of a high joint commission to treat with the Medical Society of the State of New York on differences which have been well known to exist for a decade, surely something must have happened to change the spirit prevailing in the rank and file which now attend this venerable and ever-to-be venerated medical organization. But more than this, a committee to revise the code of ethics was appointed, which, if it performs its duties in the line and direction that is confidently to be expected, will lead to further progress in the reconciliation of differences that were heretofore thought to be insurmountable. Taken altogether, then, we may affirm that the remark made at the opening of this article is justified, namely, that the forty-third meeting of the American Medical Association, at Detroit, was a remarkable one,—remarkable for progress and improvement in substantial and needed directions.

